# In Vitro and In Vivo Evaluation of Voriconazole-Containing Antifungal Combinations against Mucorales Using a *Galleria mellonella* Model of Mucormycosis

**DOI:** 10.3390/jof5010005

**Published:** 2019-01-08

**Authors:** Daiana Macedo, Florencia Leonardelli, Catiana Dudiuk, Roxana G. Vitale, Eleodoro Del Valle, Gustavo Giusiano, Soledad Gamarra, Guillermo Garcia-Effron

**Affiliations:** 1 Consejo Nacional de Investigaciones Científicas y Tecnológicas CONICET, Laboratorio de Micología y Diagnóstico Molecular—Cátedra de Parasitología y Micología—Facultad de Bioquímica y Ciencias Biológicas—Universidad Nacional del Litoral, Santa Fe CP 3000, Argentina; daiana.magalimacedo@gmail.com (D.M.); leonardellifs@gmail.com (F.L.); cbdudiuk@gmail.com (C.D.); 2 Unidad de Parasitología, Laboratorio de Micología, Hospital Ramos Mejía, CONICET, Ciudad Autónoma de Buenos Aires CP1221, Argentina; Roxana_vitale@yahoo.com; 3 Facultad de Ciencias Agrarias, Universidad Nacional del Litoral, Esperanza CP3080, Argentina; eleodoro77@gmail.com; 4 Consejo Nacional de Investigaciones Científicas y Tecnológicas CONICET, Área Micología, Instituto de Medicina Regional, Universidad Nacional del Nordeste, Resistencia CP3500, Argentina; gustavogiusiano@yahoo.com.ar; 5 Laboratorio de Micología y Diagnóstico Molecular—Cátedra de Parasitología y Micología—Facultad de Bioquímica y Ciencias Biológicas—Universidad Nacional del Litoral, Santa Fe CP3000, Argentina; mgamarra@fbcb.unl.edu.ar

**Keywords:** mucormycosis, *Galleria mellonella*, synergism, azole, voriconazole, amphotericin B

## Abstract

Mucorales are resistant to most antifungals. Mucormycosis associated mortality is unacceptable and new treatment approaches are needed. The objectives of this work were (i) to evaluate the nature and intensity of the in vitro effect of three drugs combinations which included voriconazole (plus amphotericin B, posaconazole and caspofungin) against 25 strains of six different Mucorales species; (ii) to evaluate a *Galleria mellonella* mucormycosis model; and (iii) to establish if any in vitro–in vivo correlation exists. As expected, amphotericin B and posaconazole were the most active drugs when tested alone. However, species-specific differences were found. The ΣFICs varied according to the used combination. Only five strains showed synergism when voriconazole was combined with posaconazole and three strains when combined with amphotericin B. Microscopic hyphae alteration were observed for some isolates when confronted against drugs combinations. Using a *Galleria mellonella* mucormycosis model, better survival was seen in voriconazole plus amphotericin B and plus caspofungin combined treatments when compared with AMB alone for *R. microsporus*. These survival improvements were obtained using a 32-fold lower amphotericin B doses when combined with VRC than when treated with the polyene alone. These lower antifungal doses emulate the antifungal concentrations where the microscopic hyphae alterations were seen.

## 1. Introduction

Mucormycosis is a rapidly progressing fungal infection with high morbidity and mortality rates. It is considered a therapeutic challenge since Mucorales are resistant to most antifungals. For many years, amphotericin B (AMB) has been the only available drug to treat these mycoses. Later, posaconazole (PSC) and more recently isavuconazole were added to the antifungal armamentarium as step-down and salvage therapy [1,2]. Despite these advances, mortality is still unacceptable and, clearly, new approaches are needed [3]. These facts have stimulated the interest in studying combinations of antifungal agents to determine if they could achieve better results. Spellberg et al. described the reasons for studying a particular combination therapy for Mucormycosis [4], including agents already approved for use in humans, which improve survival in animal models and known to be effective and safe. Following these premises, several reports were published with variable results. Most of them, describing the effectiveness of different combinations assessed empirically (tested in vivo without a previous in vitro evaluation) using murine models of infection. These studies included combinations of AMB with different echinocandins and/or iron chelators and the combination of azole (in vitro active against Mucorales as isavuconazole and PSC) with echinocandins and calcineurin inhibitors [5,6,7,8,9,10,11]. However, most of these studies do not include species and/or variety identifications, nor do they use drug combinations previously evaluated in vitro nor have combinations that include voriconazole been tested. The objectives of this work were (i) to evaluate the nature and the intensity of the in vitro effect of drug combinations including voriconazole (VRC) plus AMB, PSC, and caspofungin (CSF) against six different Mucorales species; (ii) to evaluate a *Galleria mellonella* model of mucormicosis; and (iii) to determine if any in vitro–in vivo correlation exists.

## 2. Materials and Methods

### 2.1. Fungal Isolates

Twenty-five Mucorales isolates, including 15 *Rhizopus microsporus* (12 formerly considered as var. *rhizopodiformis* and 3 as var. *microsporus*) [12], 3 *Rhizopus oryzae*, 4 *Syncephalastrum racemosum*, 1 *Lichthemia corymbifera*, 1 *Lichthemia blakesleeana*, and 1 *Lichethemia ramosa*, were studied. Of those, 19 strains have clinical origin including rhinocerebral mucormycosis (*n* = 9), osteomyelitis (*n* = 5), surgical wound infection (*n* = 3), cutaneous ulcer (*n* = 1) and one with no isolation site data (Table 1). *Candida parapsilosis sensu stricto* ATCC 22019 and *C. krusei* ATCC 6258 were used as control isolates for susceptibility testing. Strains were identified by morphological, physiological and molecular criteria. The morphology and the relative position of the strain’s sporangium, aphophysis and columella were analyzed [13,14,15]. Molecular identification was done by a PCR-RFLP [16] and by internal transcribed spacer 1 and 2 regions (ITS1 and ITS2) sequencing [17,18,19]. Thermotolerance (radial growth measurement after 120 h of incubation at 50 °C) was used to differentiate *R. microsporus* former varieties since molecular methods are unable to do so [14,15].

### 2.2. Susceptibility Testing for Individual Drugs and Combination of Drugs

The MICs values of VRC, AMB, CSF, and PSC (all obtained from Sigma-Aldrich, Buenos Aires, Argentina) were obtained following the CLSI M38 3rd edition protocol [20]. Drug interactions were evaluated by a two-dimensional checkerboard microdilution technique using the drug concentration ranges, inoculum, media, and incubation conditions of the same CLSI protocol [20]. The nature and intensity of drugs interactions were quantitatively evaluated using the fractional inhibitory concentration index (FIC index), which was defined as follows: ∑FIC-MIC = FIC A + FIC B = (MIC of drug A in combination/MIC of drug A alone) + (MIC of drug B in combination/MIC of drug B alone). Interpretive criteria used to define the interactions by using MICs endpoints were: synergism, ΣFIC ≤ 0.5; no interaction, ΣFIC > 0.5 but ≤4.0 and antagonism, ΣFIC > 4.0 [21].

### 2.3. Galleria mellonella Mucormycosis Model for the Evaluation of the Effect of Antifungal Drugs Combinations

*G. mellonella* caterpillars in their final stage of larval development (Provided by the Agricultural Sciences School—Universidad Nacional del Litoral, Argentina) were used. Larvae were classified by body weight and divided into groups of 10 individuals weighting 250 ± 50 mg. In order to establish the appropriate inoculum able to cause, at least 80% mortality at 48 h (consistent with recorded mortality in humans) [22] different spore concentration of *R. microsporum* LMDM-165 and *R. oryzae* LMDM-597 were tested. Inocula concentrations were determined by spore counting in a hemocytometer chamber, adjusted in phosphate-buffered saline (PBS) and confirmed by post infection viable counts. Groups of 10 caterpillars were infected with 10 µL of the different inoculum suspensions of each strain, resulting in 10^3^, 10^4^, 10^5^, 10^6^, and 10^7^ spores/larva. Hamilton syringes (Fisher Scientific, Buenos Aires, Argentina) were used to inject spore suspensions into *G. mellonella*´s hemocoel through the last left proleg. Control groups of 10 caterpillar each were added including an untouched group, a pierced group and an injected with 10 µL of PBS group. Larvae were incubated up to seven days at 37 °C, and survival was recorded daily. End points were considered when larvae did not respond to physical pressure. The antifungal treatment model was established by replicating the described experiments but modifying the number of doses of AMB and adding a toxicity control group. We chose AMB to test the survival increase (comparing with infection control) since this polyene is one of the antifungals of choice to treat Mucormycosis [23] and the most used in South America to treat these mycoses. Preparation of antifungal agents for injection, used doses and tolerability of the treatment in larva of *G. mellonella* were done following the protocol described by Kloezen et al. [24]. The number of doses necessary to improve the survival at 96 h post-infection was assessed by evaluating the effect of one, two or three drug doses over the infected caterpillar within 2 h, 26 h, and 50 h post infection also following the procedure described before [24]. Ten µl of each of the tested drug solution, which resulted in a final concentration of 1 mg/kg for AMB and CSF and 10 mg/kg for VRC and PSC were inoculated into the last right proleg of each caterpillar. The used concentrations are equivalent to the therapeutic concentrations [23]. The *G. mellonella* groups which received combined treatments were inoculated with 10 µL of a combined drug solution using the lowest antifungal doses where hyphae alterations (HA) were observed (more details in the result section). These doses were used to test the impact of the HA in an in vivo model. The reduction on the tested concentrations were in accordance with the results obtained for *R. microsporus* LMDM-165 in the in vitro synergism experiments (more detail in the Results section). Experiments were performed in triplicate and repeated three different days with similar results using 10 *G. mellonella* caterpillar per tested condition (total *n* = 30). These experiments include inoculum establishment, doses, toxicity, single-drug, and combination treatments.

### 2.4. Data Analysis

In vitro susceptibility (MICs and ΣFICs) and in vivo data are the results of experiments performed in triplicate and on three separate days. Continuous variables (ΣFICs) are expressed as arithmetic means while geometric means (GMs) were used to statistically compare MIC results. The off-scale MICs were converted to the next concentration up or down and were included in the analysis. To establish susceptibility differences between strains, MICs values were approximated to a normal distribution by transforming them to log_2_ values. The significance levels of susceptibilities differences were determined by Student’s *t*-test (unpaired, unequal variance). *P* values ≤ 0.05 were considered significant. Kaplan–Meier survival analysis was used to compare the time of death for each group of caterpillars in the *G. mellonella* infection and treatment models. *P* values were calculated by the log rank (Mantel-Cox) test. A *P* value ≤ 0.05 was considered significant. Statistical analyses were performed with GraphPad Prism, version 7.03 (GraphPad Software, Inc., La Joya, CA, USA).

## 3. Results

### 3.1. Individual Drug and Combined Susceptibility Testing

The in vitro activities of the tested antifungal drugs are summarized in Table 1. As expected, AMB and PSC were the most active drugs (both drugs showed GM 1.03 mg/L for all isolates). However, when *R. microsporus* (*n* = 15) MICs were compared with those of other Mucorales isolates (*n* = 10) species-specific differences were found for AMB but not for PSC. For the polyene, *R. microsporus* showed statistically higher MICs than the other species (1.45 mg/L and 0.62 mg/L, respectively; *P* = 0.0091). Elevated VRC and CSF MICs were obtained for all the isolates although slightly significant lower VRC MIC GMs were observed for *R. microsporus* than for the other Mucorales (4.59 mg/L vs. 8.00 mg/L, respectively; *P* = 0.0295). The ΣFICs varied according to the used combination. Briefly, for VRC + PSC only four strains and for VRC + AMB three strains showed ΣFIC = 0.5 (borderline synergism) for each combination and only one *L. ramosa* strain showed ΣFIC = 0.31 for VRC + PSC combination. The VRC + CSF combination exhibited ΣFIC interpreted as having no interaction for all the tested strains with ΣFIC values averaging 1.45.

**Figure 1:**
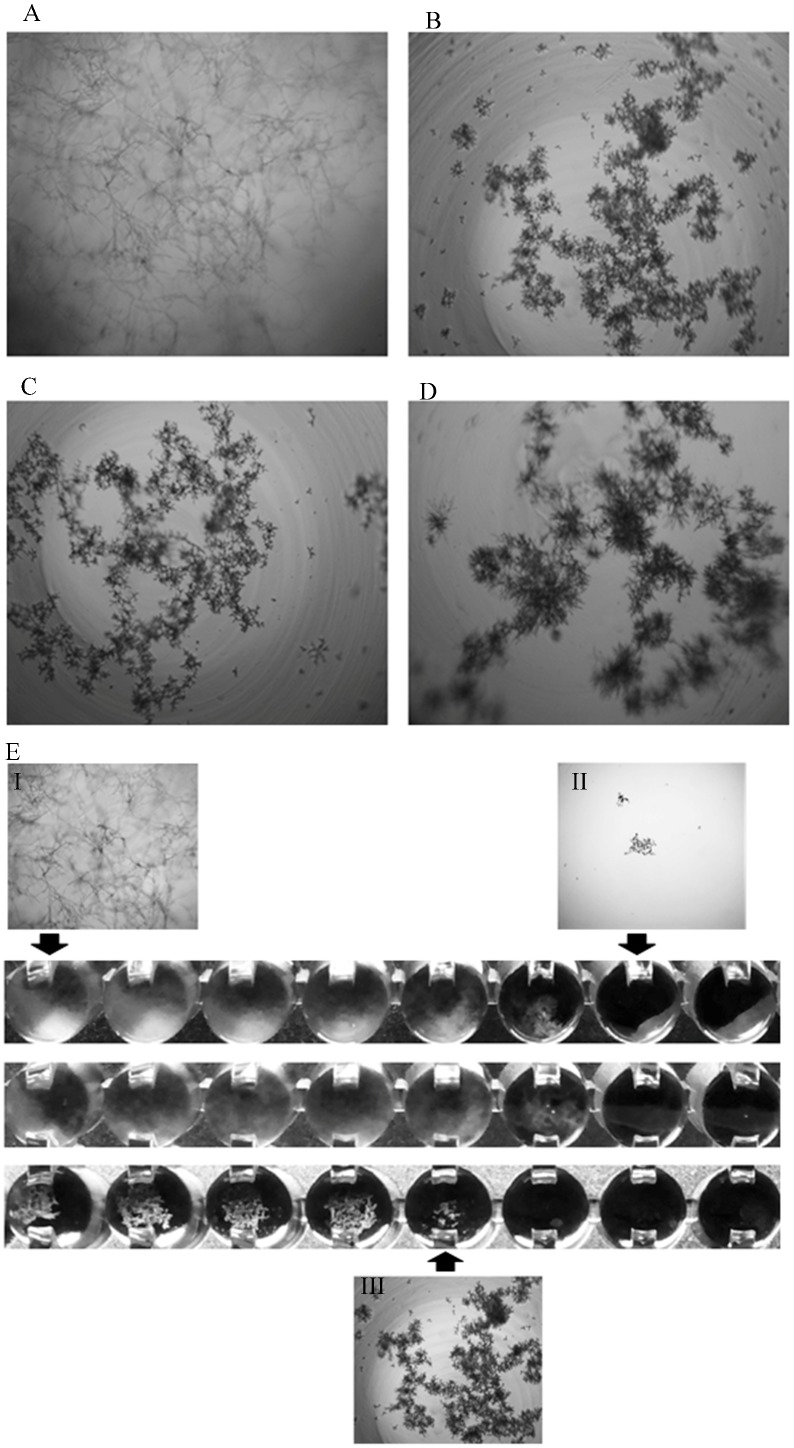
Light microphotography (50×) of morphology changes in *R. microsporus* LMDM-165 observed in combinations of drugs. (**A**) Growth control; (**B**) VRC + AMB (2 mg/L + 0.06 mg/L, respectively); (**C**) VRC + PSC (2 mg/L + 0.25 mg/L, respectively); (**D**) VRC + CSF (2 mg/L + 4 mg/L, respectively). (**E**) Microdilution plates showing AMB alone (upper line: 0.03–4.00 mg/L), VRC alone (middle line, 0.12–16.00 mg/L) and VRC + AMB combination (lower line). AMB and VRC MICs are 2 mg/L and 8 mg/L, respectively. The corresponding microscopic appearance are shown (I: growth control, II: 100% inhibition and III: Microscopic hyphal alterations).

Microscopic HA (defined as small, rounded, compact hyphal forms, as compared to the growth control) were observed for some isolates when confronted against VRC + AMB, VRC + PSC and VRC + CSF combinations (Figure 1). This effect resembles the minimal effective concentration (MEC) reported for filamentous Ascomycetes growing in the presence of an echinocandin drug [20,25]. It was observed in 48% (12 out of 25 strains), 28% (*n* = 7) and 36% (*n* = 9) of the tested strains when confronted against VRC + PSC, VRC + AMB, and VRC + CSF combinations, respectively (Table 1). No microscopic HA were seen for any of the tested strains when individual drugs were tested.

### 3.2. Galleria mellonella Infection and Antifungal Treatment Model

**Figure 2:**
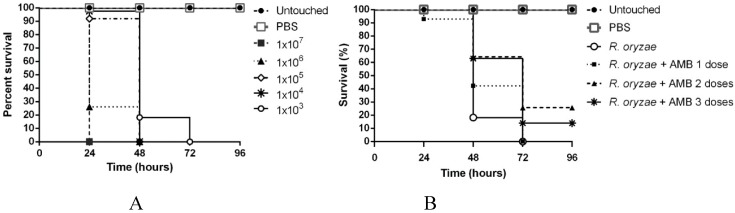
Survival curves used to establish the *Galleria mellonella* model of mucormycosis. (**A**) Infection model establishment by using different *R. oryzae* LMDM-597 inocula; (**B**) Mucormycosis treatment model using *R. oryzae* and different number of AMB doses (1 mg/kg/day).

**Figure 3:**
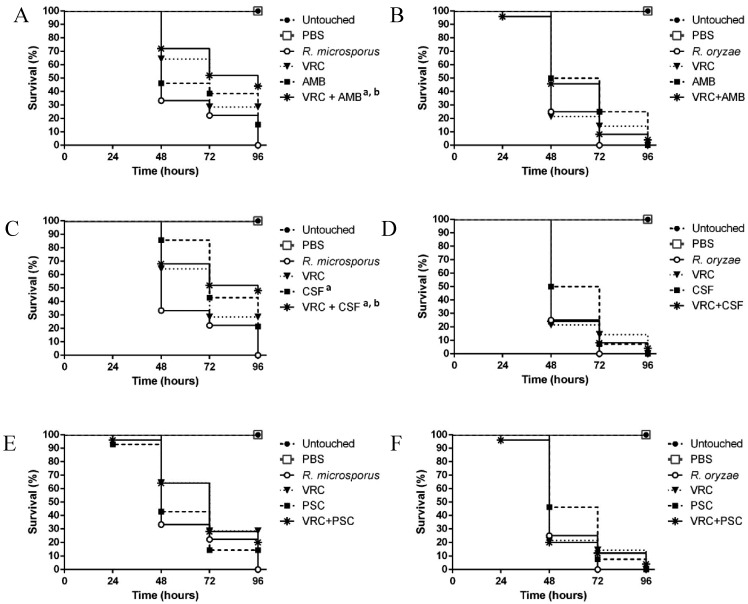
Survival curves of *G. mellonella* infected with *R. microspores* LMDM-165 treated with two doses of: (**A**) VRC (10 mg/kg/day), AMB (1 mg/kg/day) and VRC + AMB (2.5 mg/kg/day + 0.03 mg/kg/day); (**C**) VRC (10 mg/kg/day), CSF (0.5 mg/kg/day) and VRC + CSF (2.5 mg/kg/day + 0.125 mg/kg/day); (**E**) VRC (10 mg/kg/day), PSC (10 mg/kg/day) and VRC + PSC (2.5 mg/kg/day + 2.5 mg/kg/day). Infections with *R. oryzae* LMDM-597 treated with: (**B**) VRC (10 mg/kg/day), AMB (1 mg/kg/day) and VRC + AMB (2.5 mg/kg/day + 0.03 mg/kg/day); (**D**) VRC (10 mg/kg/day), CSF (0.5 mg/kg/day) and VRC + CSF (2.5 mg/kg/day + 0.125 mg/kg/day) (**F**) VRC (10 mg/kg/day), PSC (10 mg/kg/day) and VRC + PSC (2.5 mg/kg/day + 2.5 mg/kg/day). ^a^ Statistically significant improvement in survival when compared with untreated. ^b^ Statistically significant improvement in survival when compared with AMB.

*G. mellonella* mucormycosis model was used to evaluate if the observed microscopic HA have any in-vivo impact. To do so, we choose a *R. microsporus* (LMDM-165) and a *R. oryzae* (LMDM-597) as infective strains, as they showed the exact MIC and ΣFIC values for all the individual drugs and combinations but different microscopic behavior when confronted against the tested drugs combinations. *R. microsporus* LMDM-165 showed ΣFICs interpreted as having no interaction but showed the mentioned microscopic HA for all the tested combinations. On the other hand, *R. oryzae* LMDM-597 exhibited the same ΣFICs but no HA were observed. The selected inoculum for the mucormycosis model was 10^3^ spores/larvae of both strains since 80% mortality was obtained at 48 h post-challenge (higher spore concentrations produced 100% mortality between the first and second day post-infection) (Figure 2). The number of AMB doses necessary to increase the survival at 96 h post-infection was assessed. A significant increase in survival was observed when comparing one and two AMB doses (*P* = 0.033) while no survival changes were seen when three AMB doses were used (*P* = 0.161) (Figure 2 and Figure 3). Thus, two doses was chosen as the ideal treatment model to be used. The same number of doses was used for all the tested treatments. Toxicity control groups showed a 100% survival (data not shown in order to reduce the complexity of Figure 2).

### 3.3. Evaluation of the Efficacy of the Drug Combinations in the Galleria mellonella Model

The median survival (days in which 50% survival was recorded) was one or two days in the untreated control groups infected with LMDM-165 and LMDM-597 strains. When individual drugs (at therapeutic doses) were used, no statistically significant improvement in survival was observed except for the LMDM-165 infected group when treated with CSF (P = 0.035) (Figure 3). Turning to drug combinations, caterpillars infected with *R. microsporus* and *R. oryzae* received a combination of drugs in doses equal to the lowest concentrations where the HA were observed in vitro for the *R. microsporus* LMDM-165 isolate. Thus, AMB doses were reduced 32 times (0.03 mg/kg/day) compared to the usual therapeutic dose. AMB MIC for both strains were 2 mg/L but the microscopic HA was observed at 0.06 mg/L when AMB was combined with other antifungals. Similarly, CSF and azole doses were reduced four times (0.125 mg/kg/day and 2.5 mg/kg/day, respectively). For these last antifungals, the HA were observed in wells where the drug concentrations were 4-fold lower than the recorded MICs, respectively. Despite these dose reductions, the median survivals for combinations were higher than for individual drugs for all the tested combinations when the infective agent was *R. microsporus* LMDM-165 (Figure 3).

These survival improvements where statistically significant when VRC + AMB and VRC + CSF treatments were compared with untreated and AMB treatment group (*P* = 0.0087 and *P* = 0.0081 vs. untreated and *P* = 0.040 and *P* = 0.045 vs. AMB treatment group, respectively). On the contrary, when *R. oryzae* infected larvae were treated with combinations, no survival improvement was seen compared to the untreated or the individual drugs groups (all the *P* > 0.05) (Figure 3). None of the drugs alone or in combinations showed toxic effect on the larvae at the used concentrations (data on the toxicity groups are not shown).

## 4. Discussion

Mucormycosis mortality rate has remained over 40% for years [22,26,27]. Effective treatment of these mycoses depends on quick diagnosis, surgical debridement, reducing or eliminating risk factors and early antifungal treatment [27]. One of the main advances in mucormycosis treatment was the shift from AMB deoxycholate to AMB lipid formulations [6,28,29]. Also, echinocandins and combination treatments were tested with promissory results when using animal models but less encouraging outcomes were obtained in clinical scenarios [3,6,30]. In 2014, Kontoyiannis et al. published a prospective clinical trial where monotherapy (mainly lipid-based AMB) was used in 60% of the cases while combination therapy (AMB+PSC and AMB+echinocandins) was used in the remaining patients. They concluded that it is still more important a prompt treatment initiation to improve survival than using a single or combination therapy [3].

Most of the published reports where treatment effectiveness was evaluated did not identify all the strains to species or variety level [3,9,31]. Our data suggest that in vitro and in vivo susceptibility to a particular treatment depends largely on the etiologic agent. We found different behavior even in the different former varieties of *R. microsporus*. These results are in accordance with previously reported data [32].

Turning to the in vivo testing of drug combinations, several reports demonstrated its effectiveness using murine models of mucormycosis due to *R. oryzae* (Gebremariam et al. also used *Mucor circinelloides*) [5]. However, none performed a previous evaluation of the in vitro activity of the tested combinations [5,6,7,8,10,11]. In this work, we firstly evaluated the in vitro activity of the tested combination and later tested them in a *Galleria mellonella* model of mucormycosis using two *Rhizopus* species with identical MIC and ΣFIC values but with different microscopic morphology after drug exposure. We used an infection model similar to the one published by Kaerger et al. [33] but using a higher incubation temperature (37 °C vs. 30 °C) and a lower inocula (10^3^ spores/larvae vs. 10^6^ spores/larvae). The rationale of these changes was the fact that most of the published models of fungal infections used 37 °C as incubation temperature [34,35,36,37,38]. However, as described by Kaerger et al. [33], thermotolerant *Rhizopus* species (human pathogens) spread faster within the caterpillar at higher temperature. Thus, inocula were reduced in our model to circumvent this issue and allow us to evaluate differences in treatment efficacy.

We decided to test combinations including VRC (VRC + CSF, VRC + AMB, and VRC + PSC) since these combinations partially fulfil what Spellberg et al. considered as characteristics that drugs combinations may have to be studied as potential options of mucormycosis treatment [4]. Thus, these drugs are agents already approved and with good efficacy and safety. However, there is no data regarding survival improvement in animal models of mucormycosis. In this work, the tested combinations improve caterpillar survival in a strain depending manner (higher survival in *R. microsporus* than in *R. oryzae* infected larvae). We also chose to test combinations of drugs with VRC based in reports where combinations that included azole drugs showed to be synergistic against azole resistant fungi [39,40,41] and that the pre-exposure to triazoles enhanced AMB activity against *Rhizopus* spp. [42]. The results shown here demonstrate that combinations of drugs that included VRC (+ AMB and + CSF) produce a morphological change in the hyphae of some Mucorales strains (not observed when treated with the antifungals alone). When these morphological modifications were observed, a better survival of *G. mellonella* caterpillars was obtained. Thus, it seems that microscopically observed HA correlates better with survival improvement than ΣFICI. The substantial reduction, especially in AMB doses, would signify a concomitant reduction in toxicity in a hypothetical combined treatment. Moreover, considering that some authors showed that Mucormycosis should be taken into account in patients receiving VRC as Aspergillosis prophylaxis [43,44,45], VRC plus a low AMB dose should be studied as a preemptive treatment option for both Aspergillosis and Mucormycosis, especially in immunosuppressed patients with diabetes and/or malnutrition [43].

When using VRC + CSF in the *G. mellonella* model, a survival improvement was also seen. It is difficult to explain how VRC is able to improve the performance of AMB and CSF when combined. However, earlier reports have demonstrated that CSF enhances the activity of other drugs against intrinsic echinocandin resistant fungi as *Cryptococcus* spp. and *Fusarium* spp. [46,47]. Similarly, combinations that included azole drugs showed to be synergistic against azole resistant fungi [39,41,48]. On the contrary, a recent work demonstrated that micafungin did not enhance the activity of isavuconazole in a murine model of mucormycosis [5]. However, this lack of improvement in the survival rate would be also related with the limited efficacy seen for this newly commercialized azole reported by Maurer et al. using a *G. mellonella* model of mucormycosis [49].

This work would be a starting point to understand how antifungal combinations alter Mucorales hyphae and contribute to *G. mellonella* survival improvement. However, further work is necessary to confirm the clinical applicability of our observations, to evaluate the relative concentrations and the pharmacodynamics of the tested antifungals within caterpillar tissues and to clarify the molecular mechanism involved in the alteration of Mucorales hyphae.

## Figures and Tables

**Table 1:** Antifungal susceptibility, ΣFIC indexes, and microscopic alteration of hyphae in combination wells of the studied Mucorales isolates

Isolate Nº	Organism	Isolation Site	MIC (mg/L)				∑FIC Index (Interpretation) ^c^					
							VRC + PSC		VRC + AMB		VRC + CSF	
			VRC	PSC	AMB	CSF	MIC	HA	MIC	HA	MIC	HA
LMDM-165	*R. microsporus* ^a^	Osteomyelitis	8.00	1.00	2.00	16.00	0.75 (NI)	Yes	0.75 (NI)	Yes	0.75 (NI)	Yes
LMDM-156	*R. microsporus* ^a^	Osteomyelitis	4.00	1.00	2.00	16.00	1.00 (NI)	Yes	2.04 (NI)	No	1.00 (NI)	Yes
LMDM-157	*R. microsporus* ^a^	Surgical wound	4.00	0.50	1.00	16.00	0.50 (S)	Yes	1.03 (NI)	No	0.75 (NI)	Yes
LMDM-158	*R. microsporus* ^a^	Surgical wound	8.00	0.50	2.00	16.00	1.00 (NI)	Yes	1.04 (NI)	No	2.00 (NI)	Yes
LMDM-159	*R. microsporus* ^a^	Surgical wound	4.00	1.00	2.00	16.00	1.00 (NI)	No	1.03 (NI)	No	1.50 (NI)	No
LMDM-164	*R. microsporus* ^a^	Osteomyelitis	4.00	1.00	2.00	16.00	1.00 (NI)	No	1.01 (NI)	Yes	1.00 (NI)	No
LMDM-167	*R. microsporus* ^a^	Hospital environment	4.00	1.00	2.00	16.00	1.02 (NI)	Yes	1.03 (NI)	No	1.25 (NI)	No
LMDM-168	*R. microsporus* ^a^	Hospital environment	4.00	1.00	2.00	16.00	1.03 (NI)	No	2.50 (NI)	No	2.00 (NI)	No
LMDM-176	*R. microsporus* ^a^	Osteomyelitis	8.00	1.00	2.00	16.00	0.63 (NI)	Yes	2.25 (NI)	Yes	1.25 (NI)	No
LMDM-184	*R. microsporus* ^a^	Osteomyelitis	8.00	1.00	2.00	16.00	0.75 (NI)	Yes	1.03 (NI)	Yes	1.03 (NI)	No
LMDM-379	*R. microsporus* ^a^	Rhinocerebral	8.00	1.00	4.00	16.00	0.63 (NI)	Yes	1.00 (NI)	No	0.75 (NI)	Yes
LMDM-596	*R. microsporus* ^a^	Rhinocerebral	2.00	1.00	1.00	16.00	1.06 (NI)	No	2.00 (NI)	No	1.03 (NI)	No
LMDM-1073	*R. microsporus* ^b^	Rhinocerebral	2.00	1.00	0.50	16.00	1.06 (NI)	No	1.06 (NI)	No	2.00 (NI)	No
LMDM-1074	*R. microsporus* ^b^	Rhinocerebral	2.00	1.00	0.25	16.00	0.75 (NI)	No	0.62 (NI)	No	2.00 (NI)	No
LMDM-1127	*R. microsporus* ^b^	Hospital environment	8.00	2.00	1.00	16.00	0.50 (S)	No	0.50 (S)	No	0.75 (NI)	No
*n* = 15	*R. microsporus*		4.59	0.95	1.45	16.00	0.82		1.14		1.18	
LMDM-597	*R. oryzae*	Rhinocerebral	8.00	1.00	2.00	16.00	0.75 (NI)	No	0.75 (NI)	No	0.75 (NI)	No
LMDM-1126	*R. oryzae*	Rhinocerebral	8.00	1.00	1.00	16.00	0.75 (NI)	No	0.50 (S)	No	1.00 (NI)	No
LMDM-1075	*R. oryzae*	Rhinocerebral	4.00	1.00	0.25	16.00	1.00 (NI)	No	0.62 (NI)	No	2.00 (NI)	No
*n* = 3	*R. oryzae*		6.35	1.00	0.79	16.00	0.83		0.61		1.14	
LMDM-1122	*S. racemosum*	Rhinocerebral	4.00	1.00	0.50	16.00	1.00 (NI)	Yes	1.00 (NI)	Yes	2.00 (NI)	Yes
LMDM-1123	*S. racemosum*	Hospital environment	4.00	1.00	0.25	16.00	0.75 (NI)	No	1.00 (NI)	No	2.00 (NI)	Yes
LMDM-1124	*S. racemosum*	Hospital environment	16.00	2.00	0.50	16.00	1.00 (NI)	No	1.00 (NI)	No	2.00 (NI)	No
LMDM-576	*S. racemosum*	Rhinocerebral	16.00	2.00	2.00	16.00	0.50 (S)	Yes	0.50 (S)	Yes	1.00 (NI)	Yes
*n* = 4	*S. racemosum*		8.00	1.40	0.59	16.00	0.78		0.88		1.75	
LMDM-1128	*L. blakesleeana*	Cutaneous ulcera	4.00	0.50	0.50	16.00	0.75 (NI)	Yes	1.00 (NI)	Yes	2.00 (NI)	No
LMDM-1121	*L. corymbifera*	Hospital environment	8.00	1.00	0.25	16.00	0.50 (S)	Yes	1.00 (NI)	No	2.00 (NI)	Yes
LMDM-1125	*L. ramosa*	Clinical ^d^	16.00	1.00	1.00	16.00	0.31 (S)	No	0.62 (NI)	No	1.00 (NI)	No
*n* = 3	*Lichtheimia* spp.		8.00	0.80	0.50	16.00	0.49		1.09		1.45	
*n* = 25	Mucormycetes		5.74	1.03	1.03	16.00	0.81		1.09		1.45	
*n* = 10	non-*R. microsporus*		8.00	1.15	0.62	16.00	0.73		0.80		1.70	

^a^ Formerly classified as *R. microsporus var. rhizopodiformis* (thermotolerant) [12]. ^b^ Formerly classified as *R. microsporus var. microsporus* (less thermotolerant) [12]. ^c^ HA: hyphal alterations were seen in wells where antifungals were combined. (S) synergism. (NI) No interaction. ^d^ No isolation site data available. Lines in the table highlighted in grey show the MIC Geometric means and the ∑FIC indexes arithmetic means per group of Mucorales species/genus.
